# Impact of pharmaceutical care integrated at a psychosocial intervention to reduce caregiver's burden in Alzheimer's disease or related dementias: Negative results at 18 months and difficulties to conduct PHARMAID RCT

**DOI:** 10.1016/j.conctc.2023.101146

**Published:** 2023-04-22

**Authors:** Teddy Novais, Soraya Qassemi, Philippe Cestac, Cécile McCambridge, Hélène Villars, Audrey Zueras, Bertrand Decaudin, Mathilde Dambrine, Dominique Huvent-Grelle, Jean Roche, Sylvie Schoenenburg, Denis Federico, Anne-Cécile Nier, Pierre Krolak-Salmon, Christelle Mouchoux

**Affiliations:** a Department of Pharmacy, Charpennes Hospital, Hospices Civils de Lyon, F-69100, Villeurbanne, France; b Lyon Institute for Aging, Hospices Civils de Lyon, F-69000, Lyon, France; c Research on Healthcare Performance (RESHAPE), INSERM U1290, University Lyon1, F-69000, Lyon, France; d REIPO Team, La Grave Hospital, Toulouse University Hospital, F-31000, Toulouse, France; e Department of Pharmacy, Toulouse University Hospital, F-31000, Toulouse, France; f Centre for Epidemiology and Population Health Research (CERPOP), UMR 1027, INSERM, University of Toulouse, F-31000, Toulouse, France; g Geriatric Department, Toulouse University Hospital, F-31000, Toulouse, France; h ULR 7365-GRITA-Groupe de Recherche sur Les Formes Injectables et Les Technologies Associées, University Lille, F-59000, Lille, France; i Pharmacie Institute, CHU Lille, F-59000, Lille, France; j Geriatric Department, CHU Lille, F-59000, Lille, France; k Department of Geriatric Psychiatry, CHU Lille, F-59000, Lille, France; l Clinical and Research Memory Centre of Lyon (CMRR), Charpennes Hospital, University Hospital of Lyon, F-69100, Villeurbanne, France; m Clinical Research Centre (CRC) - VCF (Aging – Brain - Frailty), Charpennes Hospital, University Hospital of Lyon, Villeurbanne, F-69000, Lyon, France; n Neuroscience Research Centre of Lyon (CRNL), F-69000, Lyon, France

**Keywords:** Pharmaceutical care, Psychosocial intervention, Caregivers, Burden, Alzheimer's disease and related dementias, Negative results

## Abstract

**Background:**

Psychosocial interventions for caregivers of patients with Alzheimer disease and relative dementias (ADRD) reported a caregiver burden improvement. Multicomponent intervention integrating pharmaceutical care has not yet been evaluated while ADRD patients and their caregivers are exposed to high risk of drug-related problems. The PHARMAID study aimed to assess the impact of personalized pharmaceutical care integrated to a psychosocial program on the burden of ADRD caregivers at 18 months.

**Methods:**

The PHARMAID RCT was conducted between September 2016 and June 2020 [ClinicalTrials.gov: NCT02802371]. PHARMAID study planned to enroll 240 dyads, i.e. ADRD patients and caregivers, whose inclusion criteria were: outpatient with mild or major neurocognitive disorders due to ADRD, living at home, receiving support from a family caregiver. Three parallel groups compared a control group with two interventional groups: psychosocial intervention and integrated pharmaceutical care at a psychosocial intervention. The main outcome was the caregiver burden assessed by the Zarit Burden Index (ZBI, score range 0–88) at 18 months.

**Results:**

Overall, 77 dyads were included (32% of the expected sample size). At 18 months, the mean ZBI scores were 36.7 ± 16.8 in the control group, 30.3 ± 16.3 for the group with psychosocial intervention, and 28.8 ± 14.1 in group with integrated pharmaceutical care at psychosocial intervention. No significant difference was demonstrated between the three groups (p = 0.326).

**Conclusions:**

The findings suggest that PHARMAID program had no significant impact on caregiver burden at 18 months. Several limitations have been highlighted and discussed by the authors in order to formulate recommendations for further research.

## 1 Introduction

Alzheimer's Disease and Related Dementias (ADRD) cause progressive cognitive and functional decline [1] and may have a significant impact on care cost [2]. Caring for patients with ADRD is accompanied with a caregiver burden that increases with the progression of the disease [3]. This burden can have physical, psychological, emotional, social and financial impact on the informal caregivers who are often represented as hidden secondary patients [4]. They frequently experience a higher risk of developing mood disorders as depression, anxiety, sleep disorders and a lower quality of life associated with a greater use of psychotropic drugs [[5], [6], [7]]. They also incur higher risk of hypertension and heart disease, decreased immunity and higher mortality [8,9]. The increasing frailty of the caregiver is a predictor of an early institutionalization of the patient over time [10]. To prevent caregiver burden, many studies have assessed the effectiveness of non-pharmacological interventions and showed a moderate improvement on caregiver burden [[11], [12], [13]]. These previous studies highlighted that psychosocial intervention is the type of intervention with the largest impact on caregiver burden.

In the PIXEL study, the mean age of the men caregivers was 73.9 years and 64.8 for the women caregivers [14]. Old age people themselves, especially spouses, caregivers are also exposed to common chronic diseases and associated polypharmacy with a higher risk of developing drug-related problems (DRPs) due to aging and negligence of their own health care (e.g. delaying care). These risks are increased in older people mainly because of changes in pharmacokinetic and pharmacodynamic parameters related to aging, acute or chronic diseases and the prescription of Potentially Inappropriate Medications (PIMs) [15]. PIMs are frequently prescribed in community-dwelling older people [16,17], and are associated with a higher morbidity, mortality, use of care and costs [18]. In addition to age-related comorbidities, the underlying disease and the associated polypharmacy, patients with ADRD have a more complex drug therapy. Interventions to detect PIMs and to control DRPs seem necessary to optimize caregiver's and ADRD patient's management. In previous studies, medication review conducted by a clinical pharmacist has shown efficacy regarding DRP reduction, length of hospital stay, readmission rates, quality of life and mortality [[19], [20], [21]]. Thus, the PHARMAID program was designed to integrate a clinical pharmacist to perform medication review and counselling in a multidisciplinary psychosocial intervention with ADRD patients and their caregivers.

This study aimed to report the results of the PHARMAID RCT at 18-month follow-up. The primary objective of the PHARMAID RCT was to assess the impact of personalized pharmaceutical collaborative care integrated to a multidisciplinary psychosocial program on the burden of ADRD caregivers. The secondary objectives was to assess the impact of the program on clinical outcomes and appropriate prescription outcomes of patients with ADRD and their caregivers.

## 2 Materials and methods

### 2.1 Study design

The PHARMAID study was a multi-center RCT assessing an integrated pharmaceutical care at a psychosocial program. Three parallel groups were studied: a Control Group (CG), a Psychosocial intervention Group (PG), and an integrated Pharmaceutical care at a Psychosocial intervention Group (iPPG). Because of the intervention components, PHARMAID RCT was an unblended study. This RCT has been registered on clinicaltrial.gov since June 16, 2016 [ClinicalTrials.gov: NCT02802371]. The study protocol was funded by the French 10.13039/100009647Ministry of Health and endorse by the French Society of Clinical Pharmacy. Details of the study protocol have been previously published [22].

### 2.2 Setting and participants

The PHARMAID RCT was conducted in 3 specialized centres in the care of patient with a neurocognitive disorders of 3 French university hospitals (Lille, Lyon and Toulouse), between September 2016 and June 2020. ADRD outpatients suffering from mild or major neurocognitive disorders and their primary caregivers were eligible for inclusion. The diagnosis of ADRD was based on the clinical criteria of Alzheimer's disease [23,24], vascular dementia [25], lewy body dementia [26], frontotemporal lobar degeneration [27] and mixed dementia. We target mild to moderate stages of the disease defined by the Mini-Mental Score Examination (MMSE), with scores of 25 to 16/30. Community-dwelling dyads, i.e. patient and caregiver, were enrolled in the PHARMAID study. The caregiver was defined as a nonprofessional person living with the patient or providing support to the patient at least 10 h a week. Only caregivers with the ability to follow the study program (at the discretion of the physician) were eligible. In the initial version of the study protocol, only patients and caregivers aged over 65 years were eligible. To deal with inclusion difficulties, an amendment of the study protocol have been made in November 2017. In the second version of study protocol, caregivers aged over 55 years became eligible. Institutionalized patients, caregivers with the disease acceptance do not allow their participation and caregivers enrolled in another program to support the family caregivers were not included in the study.

### 2.3 Primary outcome

The primary outcome of the PHARMAID RCT was the caregiver burden measured by the Zarit Burden Index (ZBI) questionnaire and evaluated at 18-month follow-up. The ZBI is a subjective measure of burden that includes 22 items exploring the caregiver's perception and feelings about care situations. Each item was evaluated using a 5-point Likert scale ranging from 0 (never) to 4 (almost always), which are summed. The score range is 0–88, a higher score indicating a higher burden level [4,28].

### 2.4 Secondary outcomes

The secondary outcomes evaluated at 18-month follow-up were: 1) the caregiver's anxiety, measured by the Hamilton Anxiety Scale (HAS) [29]; 2) the caregiver's depression, measured by the Geriatric Depression Scale (GDS) [30]; 4) the patient's quality of life measured by the Alzheimer Disease Related Quality of Life (ADRQL) [31]; 5) the frequency and severity of the patient's Alzheimer Disease Related Quality of Life (BPSD) by the Neuropsychiatric Inventory (NPI) [32]; 6) the patient's functional autonomy assessed by the Instrumental Activities of Daily Living (IADL) [33]. Secondary outcomes evaluated at 18-month follow-up about the appropriateness of the dyad medication prescriptions were: 1) the number of drug prescribing; 2) the PIM prevalence according to the EU(7)-PIM list [34]; 3) the medication regimen complexity using the Medication Regimen Complexity Index (MRCI) [35].

### 2.5 Detailed study scheme

**Fig. 1:**
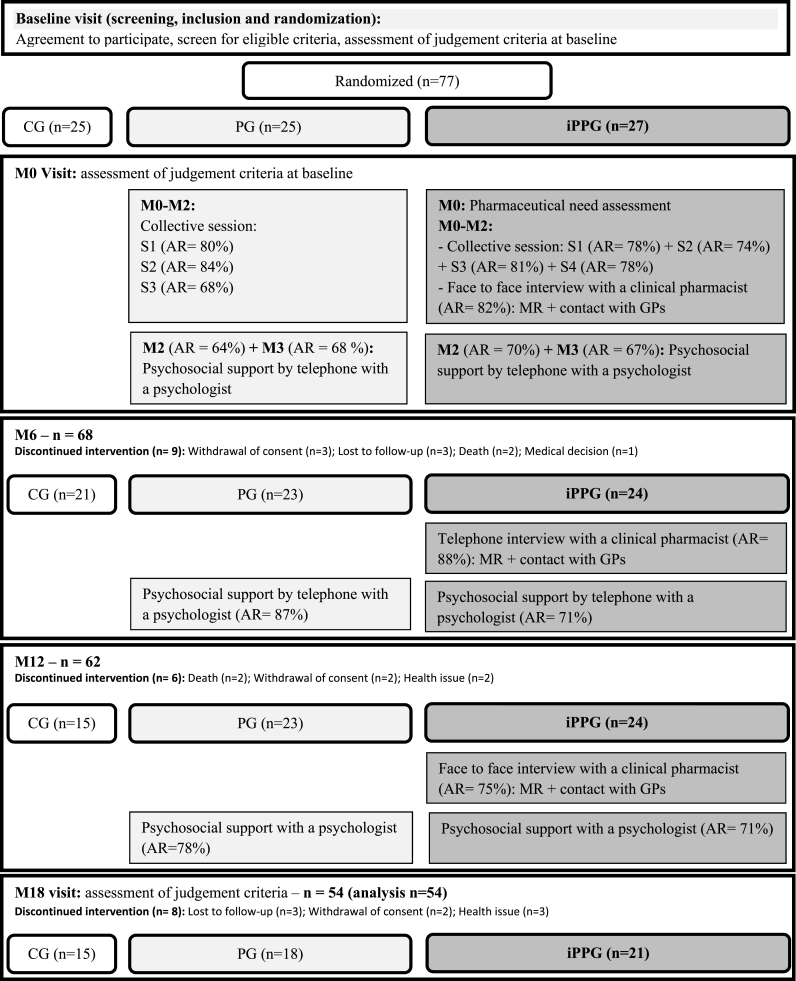
Study design and flow chart of PHARMAID study.

The baseline and follow-up procedures are illustrated in Fig. 1.

The baseline visit (M0) concerns both the patient and the caregiver and at the end, each patient/caregiver dyad is randomly allocated to CG, PG or iPPG. The random number is generated by a computerized generator using block randomization. Randomization is stratified by center and the block size consists of 9 ADRD patient/caregiver dyads to facilitate the organization of collective sessions. Patients and their caregivers have been followed during18 months.

### 2.6 Description of the control group

Patients and caregivers randomized in CG benefited from the current management without any specific psychosocial intervention or pharmaceutical care in the study centres.

### 2.7 Interventions

#### 2.7.1 Psychosocial intervention and support (PG)

Caregivers of the dyads included in the PG benefited from a multi-component intervention with three collective sessions: (S1) “Impact of the disease” by a geriatrician and a psychologist (3 h), (S2) “Living together” by a psychologist (3 h), (S3) “Local ressources” by a social worker (2 h); and, individual support with a psychologist in face-to-face or by telephone according to the follow-up time (Fig. 1). During the first month after the inclusion, caregivers participated in three collective sessions conducted in small groups (six caregivers) to encourage the interactions. Individual interviews with a psychologist were conducted by telephone (M2, M3 and M6) to assess the positive and negatives changes in these following domains: cognition, behavior, autonomy, activities, caregiver stress and well-being. These interviews also aimed to support psychologically the caregivers and to provide counselling. At M12, a face-to-face interview was conducted with the same psychologist to summarize the benefits of this intervention and to anticipate the future according to their proper needs by referring them to others programs or structures. The organization of these sessions follows the Behavioral and Cognitive Therapies Recommendations [36].

#### 2.7.2 Integrated pharmaceutical care at psychosocial intervention and support (iPPG)

Caregivers of the dyad included in iPPG benefited from the same multi-component intervention that PG with the integration of pharmaceutical care by a clinical pharmacist. The clinical pharmacist intervened in: 1) the pharmaceutical need assessment of the caregivers considering their medication management and the medication management of their relatives at the inclusion; 2) a collective session on medication management of the dyad (S4, 1.5 h); 3) personalized interviews to consider caregiver's needs, medication problems and difficulties; 4) Medication review and transmission of pharmaceutical interventions about the patient's and caregiver's prescriptions to their General Practitioners (GPs) and community pharmacists. A pharmaceutical intervention was defined as ‘any action initiated by a pharmacist directly resulting in a change of the patient's management or therapy’ [37].

### 2.8 Statistical analysis

We conducted a sample size estimation based on the existing literature [22]. The total sample size required for the study was 240 dyads (80 per arm). The SPSS v.21 was used for statistical analyses. At baseline, socio-demographic characteristics of the three groups were compared using ANOVA (quantitative variables) or Chi-square test (qualitative variables). To determine the impact of the PHARMAID program, per protocol analysis was conducted. The caregivers' and patients’ outcomes were compared at baseline and at 18 month, using ANOVA or Chi-square test. Caregiver adherence to the PHARMAID program was assessed by calculating the participation rates to each component of the PHARMAID program. The individual caregiver adherence rate was assessed by calculating the individual participation rate to components of the PG intervention (7 components) and to iPPG intervention (11 components). In this study, adherent caregivers were defined as caregivers with an individual adherence rate ≥80% to components of the PHARMAID program. A sub analysis, using Mann-Whitney test, was achieved to compare the caregiver burden at 18 month between adherent and non-adherent caregivers in PG and in iPPG. A two-sided P value less than 0.05 was considered statistically significant.

### 2.9 Ethical consideration

All participants gave their verbal informed consent after being told about the study. This study was conducted in accordance with the declaration of Helsinki. The study protocol has been reviewed and approved by the Committee for the Protection of Persons (CPP) the September 08, 2015.

## 3 Results

### 3.1 Patient characteristics

A total of 77 dyads were included in this study (32% of the expected sample size) and only 54 dyads participated until 18 month of follow-up (Fig. 1). At baseline, 71.5% of caregivers were females, 83.1% were spouses and 87.0% lived at home with the patient (Table 1). The mean age of caregivers was 77.0 ± 6.7 years (0% under 65 years despite the amendment of the study protocol). Regarding the patients in the dyads, 41.5% were females (Table 2). Their mean age was 81.1 ± 5.5 years. The main diagnosis etiology was Alzheimer's disease (71.4%) and the main diagnosis stage was dementia (76.6%). There were no significant differences between caregivers and patients of the three groups with respect to their sociodemographic characteristics as shown in Table 1, Table 2 At 18 month, 23 dyads were no longer followed (attrition rate of 30%): 10 in CG (40% of the arm), 7 in PG (28% of the arm) and 6 in iPPG (22% of the arm) (Fig. 1). The main reasons for the study discontinuation were withdrawal of consent (n = 7), lost of follow-up (n = 6), health issue (n = 5), and death (n = 4). During the study, six patients were institutionalized.

**Table 1:** Characteristics of caregivers.

Variables	Total n = 77	CGn = 25	PG n = 25	iPPG n = 27	*P* value
Age mean, Years (SD)	77.0 (6.7)	78.3 (6.6)	75.9 (8.1)	76.8 (5.4)	0.439
Females (%)	55 (71.5)	18 (72.0)	18 (72.0)	19 (70.4)	0.989
Education (%)					
Primary	12 (15.6)	4 (16.0)	2 (8.0)	6 (22.2)	0.729
Secondary	42 (54.5)	13 (52.0)	16 (72.0)	13 (48.1)	
Tertiary	18 (23.4)	7 (28.0)	4 (16.0)	7 (25.9)	
Nil	5 (6.5)	1 (4.0)	3 (12.0)	1 (3.7)	
Marital status (%)					
Married/in couple	68 (88.3)	22 (88.0)	21 (84.0)	25 (92.6)	0.550
Single/Divorced/widowed	9 (11.7)	3 (12.0)	4 (13.1)	2 (7.4)	
Relationship with the patient (%)					
Spouse	64 (83.1)	22 (88.0)	19 (76.0)	23 (85.2)	0.617
Child	10 (13.0)	2 (8.0)	5 (25.0)	3 (11.1)	
Brother/Sister	2 (2.6)	0 (0)	1 (4.0)	1 (3.7)	
Other	1 (1.3)	1 (4.0)	0 (0)	0 (0)	
Living situation (%)					
At home with patient	67 (87.0)	22 (88.0)	22 (88.0)	23 (85.2)	0.497
At home with family	4 (5.2)	0 (0)	2 (8.0)	2 (7.4)	
At home alone, without family nearby	2 (2.6)	1 (4.0)	1 (4.0)	0 (0)	
At home alone, with family nearby	4 (5.2)	2 (8.0)	0 (0)	2 (7.4)	

Legends: CG: Control group; iPPG: integrated Pharmaceutical care at a Psychosocial intervention Group; PG: Psychosocial intervention Group; SD: Standard Deviation.

**Table 2:** Characteristics of patients.

Variables	Total n = 77	CG n = 25	PG n = 25	iPPG n = 27	*P* value
Age mean, Years (SD)	81.1 (5.5)	81.7 (5.1)	80.0 (6.7)	81.5 (4.6)	0.479
Females (%)	32 (41.5)	9 (36.0)	11 (44.0)	12 (44.4)	0.790
Education (%)					
Primary	26 (33.8)	8 (32.0)	10 (40.0)	8 (29.6)	0.214
Secondary	29 (37.6)	10 (40.0)	7 (28.0)	12 (44.4)	
Tertiary	19 (24.7)	7 (28.0)	5 (20.0)	7 (25.9)	
Nil	3 (3.9)	0 (0)	3 (12.0)	0 (0)	
Marital status (%)					
Married/in couple	63 (81.8)	22 (88.0)	19 (76.0)	22 (81.5)	0.545
Single/Divorced/Widowed	14 (18.2)	3 (12.0)	6 (24.0)	5 (18.5)	
Living situation (%)					
At home with spouse	64 (83.1)	22 (88.0)	19 (76.0)	23 (85.2)	0.269
At home with family	3 (3.9)	0 (0)	3 (12.0)	0 (0)	
At home alone, without family nearby	2 (2.6)	1 (4.0)	1 (4.0)	0 (0)	
At home alone, with family nearby	8 (10.4)	2 (8.0)	2 (8.0)	4 (14.8)	
Etiological diagnosis (%)					
Alzheimer's disease	55 (71.4)	15 (60.0)	17 (68.0)	23 (85.2)	0.261
Alzheimer's disease with cardiovascular component	12 (15.6)	5 (20.0)	5 (20.0)	2 (7.4)	
Vascular dementia	4 (5.2)	3 (12.0)	0 (0)	1 (3.7)	
Lewy body disease	1 (1.3)	0 (0)	1 (4.0)	0 (0)	
Frontotemporal dementia	1 (1.3)	0 (0)	0 (0)	1 (3.7)	
Other dementia	2 (5.2)	2 (8.0)	1 (4.0)	0 (0)	
Diagnosis stage (%)					
Mild cognitive impairment	15 (19.5)	4 (16.0)	7 (28.0)	4 (14.8)	0.575
Dementia	59 (76.6)	19 (76.0)	17 (68.0)	23 (85.2)	
Missing data	3 (3.9)	2 (8.0)	1 (4.0)	0 (0)	
MMSE mean (SD)	20.0 (3.7)	19.9 (2.7)	20.1 (3.3)	20.0 (4.9)	0.981

Legends: CG: Control group; iPPG: integrated Pharmaceutical care at a Psychosocial intervention Group; MMSE: Mini Mental State Examination; PG: Psychosocial intervention Group; SD: Standard Deviation.

### 3.2 Results on primary outcome: caregiver burden

Despite the randomization process, the caregiver burden (ZBI) at baseline tented to be higher in the CG in comparison with other groups: 34.3 ± 12.9 in CG, 26.1 ± 16.0 in PG, and 26. ± 12.5 in iPPG (p = 0.060) (Table 3).

**Table 3:** Caregiver outcomes.

Outcomes	CG n = 25	PG n = 25	iPPG n = 27	*p* value
Caregiver burden, ZBI mean (SD)				
Baseline	34.3 (12.9)	26.1(16.0)	26.2 (12.5)	0.060
18 months	36.7 (16.8)	30.3 (16.3)	28.8 (14.1)	0.326
Depression, GDS mean (SD)				
Baseline	9.44 (5.2)	9.1 (5.6)	9.3 (4.6)	0.976
18 months	11.20 (6.1)	9.7 (7.7)	10.9 (6.0)	0.772
Anxiety, Hamilton mean (SD)				
Baseline	9.6 (7.7)	9.7 (8.5)	7.8 (4.1)	0.530
18 months	9.9 (7.9)	9.1 (9.2)	8.5 (5.6)	0.851
Medication number mean (SD)				
Baseline	5.5 (5.1)	4.1 (3.0)	4.4 (2.2)	0.383
18 months	5.9 (4.9)	4.2 (3.3)	4.5 (2.1)	0.357
PIM prevalence (%)				
Baseline	13 (52.0)	11 (44.0)	11 (40.1)	0.773
18 months	11 (44.0)	10 (40.0)	10 (37.0)	0.632
Medication regimen complexity, MRCI mean (SD)				
Baseline	13.6 (15.2)	9.1 (6.9)	11.0 (7.0)	0.328
18 months	12.8 (14.1)	9.1 (6.9)	12.2 (7.0)	0.485

Legends: CG: Control group; GDS: Geriatric Depression Scale; iPPG: integrated Pharmaceutical care at a Psychosocial intervention Group; PG: Psychosocial intervention Group; PIM: Potentially Inappropriate Medication; SD: Standard Deviation; ZBI: Zarit Burden Index.

At 18 months, there was no significant difference between the three groups (p = 0.326), but the caregiver burden tented to be lower in iPPG (28.8 ± 14.1) and PG (30.3 ± 16.3) in comparison with CG (36.7 ± 16.8). However, 10 patients in CG discontinued the study. A sub-analysis was performed to compare the caregiver burden at baseline between caregivers who discontinued the study and caregivers followed during 18 months. At baseline, the ZBI score was 27.0 ± 14.5 in caregivers that completed the study and 33.2 ± 12.5 for caregivers that discontinued the study (p = 0.079).

### 3.3 Results on secondary outcomes

Regarding caregiver clinical outcomes such as depression and anxiety, no significant difference was demonstrated at baseline and at 18 months (Table 3). There was also no statistically significant difference for patient clinical outcomes during the follow-up (Table 4). However, at baseline, the patients’ quality of life, the autonomy and the BPSD tended to be more impaired in the CG.

**Table 4:** Patient outcomes.

Outcomes	CG n = 25	PG n = 25	iPPG n = 27	*p* value
Quality of life, ADRQL mean (SD)				
Baseline	76.0 (13.0)	78.9 (9.4)	77.8 (10.2)	0.639
18 months	74.7 (15.3)	75.9 (13.7)	72.0 (15.2)	0.710
Autonomy, IADL mean (SD)				
Baseline	3.6 (2.5)	4.1 (2.6)	4.0 (2.2)	0.743
18 months	2.8 (1.9)	3.1 (2.7)	2.8 (2.3)	0.904
Behavior, NPI mean (SD)				
Baseline	18.6 (16.4)	15.0 (12.2)	16.4 (14.2)	0.679
18 months	18.9 (13.4)	17.6 (13.4)	12.8 (16.5)	0.408
Medication number mean (SD)				
Baseline	7.2 (2.8)	6.2 (2.3)	7.1 (2.5)	0.348
18 months	6.7 (3.2)	5.9 (2.3)	6.6 (3.1)	0.663
PIM prevalence (%)				
Baseline	17 (68.0)	14 (56.0)	13 (48.1)	0.622
18 months	9 (36.0)	12 (48.0)	13 (48.1)	0.755
Medication regimen complexity, MRCI mean (SD)				
Baseline	16.4 (8.1)	14.2 (6.1)	18.2 (9.6)	0.242
18 months				
18 months	16.1 (9.4)	13.9 (7.8	16.8 (8.6)	0.583

Legends: ADRQL: Alzheimer Disease Related Quality of Life; CG: Control group; IADL: Instrumental Activities of Daily Living; iPPG: integrated Pharmaceutical care at a Psychosocial intervention Group; NPI: Neuropsychiatric Index; PG: Psychosocial intervention Group; PIM: Potentially Inappropriate Medication; SD: Standard Deviation.

Regarding medication outcomes at baseline, caregivers used 4.7 ± 3.7 medications and patients used 6.8 ± 2.6 medications (Table 3, Table 4), the PIM prevalences was 60.3% in patients and 48.6% in caregivers, and the mean MRCI was 16.3 ± 8.1 for patients and 11.3 ± 10.5 for caregivers. There was no significant difference in medication number, PIM prevalences and medication regimen complexity, for caregivers and patients, between the three groups at baseline and at 18 months.

### 3.4 Adherence to PHARMAID program

The mean adherence rates to the PHARMAID intervention were 76% in PG and 76% in iPPG. The adherence rates of each component of the PHARMAID program are presented in Fig. 1. A sub analysis of the caregiver burden (ZBI) at 18 months between adherent caregivers and non-adherent caregivers showed no significant difference. In PG, the ZBI in non-adherent caregivers (n = 4) was 35.0 ± 16.5 and in adherent caregivers (n = 14), the ZBI was 29.3 ± 16.7 (p = 0.487). In iPPG, the ZBI in non-adherent caregivers (n = 5) was 29.6 ± 11.5 and in adherent caregivers (n = 16), the ZBI was 28.6 ± 15.1 (p = 0.679).

## 4 Discussion

The PHARMAID program was designed to decrease the caregiver burden by transmitting knowledge, expertise and coping skills to the ADRD caregivers. The study also aimed to assess the impact of the clinical pharmacist integration in the multidisciplinary team involved in the management of patients with neurocognitive disorders and their caregivers. In the PHARMAID study, 240 dyads had to be included for the efficacy assessment. However, only 77 dyads were included related to inclusion difficulties which will be discussed.

Behind the difficulties to include dyads, the investigators of the study centres (physicians and pharmacists) communicated during regular meetings to identify the inclusion difficulty reasons. The main identified difficulties were.

1) The difficulty for caregivers, especially spouses, to be available to participate in collective sessions and to leave home alone their relative. Moreover, the majority of the included patients were at the dementia stage, which increased this difficulty;
2) The caregiver's age criterion for inclusion of 65 years and over, mainly targeting spouses with health problems themselves and also increasing the first identified difficulty. This age criteria had been chosen to perform caregiver medication review using geriatric tools such as the EU-PIM list;
3) Unlike the included patients, caregivers of dyads were not necessarily patients of the hospitals of study centres. Thus, it was more difficult to collect medical data, and to communicate information to their GPs;
4) The costs for caregivers (travel and meal expenses) to participate in collective sessions.

To cope with these difficulties, two amendments of the study protocol have been made in February and November 2017. The substantial changes to the protocol aimed to extend the inclusion period; to add a fixed allowance of 70 euros for the caregivers’ participation in collective sessions (travel and meal expenses); and to modify the minimum age criterion for caregivers by lowering it to 55 years old instead of 65 years old. However, no dyads with under 65 years caregivers were included after the amendment and difficulties to include dyads remained. Other difficulties linked to the inclusion period extension have been identified by investigators such as: the appearance of new and concurrent studies and, the difficulty to organize collective sessions (6 caregivers) with sporadic inclusions.

The efficacy of the PHARMAID program was not demonstrated at 18 month in the PG and the iPPG. Indeed, no significant impact on caregiver burden, on other caregiver outcomes and on patient outcomes have been shown. Several hypotheses could be raised.

1) The sample size and the lack of statistical power;
2) The presence of a CG without any intervention may lead to the caregiver disappointment increasing the rate of consent withdrawal or lost to follow-up (40% of dyads included in CG were no longer followed up at 18 months);
3) The heterogeneity of the 3 groups at baseline (related to the sample size). Indeed, caregivers in CG tended to have a higher burden in comparison with caregivers in PG and iPPG. The randomization announcement in CG before the visit of outcome assessment could bias the assessment of caregiver burden, anxiety and depression symptoms;
4) The PHARMAID program adherence. The lack of statistical power did not make it possible to highlight a significant difference between adherent caregivers and non-adherents.
5) The communication difficulties between hospital and community professionals: suboptimal consideration of pharmacist's recommendations in iPPG;
6) The impact of the disease progression on the caregiver burden during an extended 18-month follow-up period, which may reduce the initial positive effect of the PHARMAID program.

Following these pitfalls, some recommendations can be formulated by the authors for further research.

1) Systematically questioning the need to include a control group without any intervention in this type of study;
2) Designing a personalized intervention integrating a need assessment to increase the caregiver motivation;
3) Considering the follow-up duration according to the neurocognitive disease severity of the included population. The 18-month follow-up of the dyad in this specific population can be questioned linked to the risk of death, institutionalization and health problems during the study;
4) Reducing the selection bias by using inclusion method to obtain a heterogeneous group of patients and caregivers regarding the disease severity and the relationship in the dyad. Indeed, clinicians tended to propose the study to the dyads that seemed most in need;
5) Limiting the travel number for caregivers and provide a financial compensation for travel and meals. Clinicians tented to propose the study to the dyads who can travel to study center for the intervention (another selection bias). Our study showed that telephone support was feasible with an acceptable adherence rate. Since the SARS-CoV-2 pandemic, Application of new technologies to medicine had a recent exponential growth. Telemedicine and telehealth has shown to be a solution for this vulnerable population and can be used to support caregivers, and to reduce travel and costs [38,39];
6) Anticipating patient occupation supervised by a professional (individual or collective) during the caregiver intervention;
7) Using telehealth to discuss about recommendations from clinical pharmacist on medication management of the dyads with the community pharmacists and GPs.

To complete these recommendations, previous systematic review and meta-analysis identified facilitators to implement interventions for ADRD caregivers [12,40]. According to the updated meta-analysis conducted by Walter et al. intervention effects on burden were greater in multicomponent interventions, in sample with younger caregivers and fewer spousal caregivers [12]. In contrast, number of sessions and setting (individual or collective) did not have significant moderating effects. Finally, a systematic review was conducted to provide information on the acceptability of psychosocial interventions for dementia caregivers [40]. Facilitators of acceptability included caregivers' need for intervention, appropriate content and organization of the intervention, and knowledge and professionalism of the health care providers. Barriers to acceptability included caregivers' poor health status, caregivers' low education level, caregiver burden, change of intervention implementers, and poor system performance of interventions [40].

## 5 Strengths of the study

The PHARMAID study allowed developing or strengthening collaboration between the study centres; between the clinical pharmacists and other health professionals involved in the care pathway of patient with neurocognitive disorders; and, between hospital and community health professionals given the pharmaceutical interventions from medication review were transmitted to GPs and community pharmacists in iPPG.

The PHARMAID study showed a high PIM prevalence in patients at baseline (60.3%) but also in caregivers (48.6%). A previous study including 57,469 older dementia patients, showed that 53.1% of patients received PIMs [41]. Regarding the PIM use in caregivers of patients with ADRD, only one study was found and showed that 39% of caregivers received at least one PIM [42]. In our study, the mean MRCI at baseline was 16.3 ± 8.1 for patients and 11.3 ± 10.5 for caregivers. A previous study established thresholds to distinguish several complexity levels: low complexity was represented by a MRCI ≤9.0, mean complexity by a 9 < MRCI ≤16.5, and high complexity by a MRCI >16.5 [43]. Considering these thresholds, ADRD patients and their caregivers included in PHARMAID study presented a moderate medication regimen complexity. No significant difference was demonstrated at 18 months regarding the patient's and caregiver's medication appropriateness between the iPPG integrating a clinical pharmacists and the other groups (PG and CG). However, the PIM prevalence and the medication regimen complexity of the dyad confirm the need of the clinical pharmacist integration to achieve collaborative medication review focusing deprescribing [44].

## 6 Conclusion

The findings of the PHARMAID study suggest that pharmaceutical care integrated in psychosocial intervention had no significant impact on caregiver burden at 18 months. Several limitations have been highlighted, including the sample size reaching only 32% of the expected dyad number. Difficulties to include caregivers and patients with ADRD were discussed and recommendations for further research were formulated by the authors and from the literature data.

## Ethics approval and consent to participate

All participants gave their verbal informed consent after being told about the study. This study was conducted in accordance with the declaration of Helsinki. The study protocol has been reviewed and approved by the Committee for the Protection of Persons (CPP) the September 8, 2015.

## Funding

This work was supported by the French Ministry of Health, grant number 14-0568 (PREPS2014).

## Authors’ contributions

TN, PC, HV, BD, DHG, DF, PKS and CM designed the PHARMAID study. TN, PC, QS, CMC, HV, AZ, BD, MD, DHG, JR, SS, DF, CAN and PKS contributed to the undertaking of the PHARMAID study. TN and CM conducted the analysis. TN drafted the manuscript with substantial input from CM. All authors were involved in the critical revision of the manuscript and approve this submitted version.

## Declaration of competing interest

The authors declare that they have no known competing financial interests or personal relationships that could have appeared to influence the work reported in this paper.

## Abbreviations

ADRD: Alzheimer disease and relative dementias
ADRQL: Alzheimer Disease Related Quality of Life
BPSD: Alzheimer Disease Related Quality of Life
CG: Control Group
DRP: Drug-Related Problems
GDS: Geriatric Depression Scale
GPs: General Practitioners
HAS: Hamilton Anxiety Scale
IADL: Instrumental Activities of Daily Living
iPPG: integrated Pharmaceutical care at a Psychosocial intervention Group
MMSE: Mini-Mental Score Examination
MRCI: Medication Regimen Complexity Index
PG: Psychosocial intervention Group
PIM: Potentially Inappropriate Medication
RCT: Randomized Controlled Trial
S1: Session1
S2: Session2
S3: Session3
S4: Session4
NPI: Neuropsychiatric Inventory
ZBI: Zarit Burden Index
